# Human Bone Marrow Mesenchymal Stem Cells Display Anti-Cancer Activity in SCID Mice Bearing Disseminated Non-Hodgkin's Lymphoma Xenografts

**DOI:** 10.1371/journal.pone.0011140

**Published:** 2010-06-16

**Authors:** Paola Secchiero, Sonia Zorzet, Claudio Tripodo, Federica Corallini, Elisabetta Melloni, Lorenzo Caruso, Raffaella Bosco, Sabrina Ingrao, Barbara Zavan, Giorgio Zauli

**Affiliations:** 1 Department of Morphology and Embryology, University of Ferrara, Ferrara, Italy; 2 Department of Life Sciences, University of Trieste, Trieste, Italy; 3 Department of Human Pathology, University of Palermo, Palermo, Italy; 4 Department of Histology and Microbiology, University of Padova, Padova, Italy; University of Barcelona, Spain

## Abstract

**Background:**

Although multimodality treatment can induce high rate of remission in many subtypes of non-Hodgkin's lymphoma (NHL), significant proportions of patients relapse with incurable disease. The effect of human bone marrow (BM) mesenchymal stem cells (MSC) on tumor cell growth is controversial, and no specific information is available on the effect of BM-MSC on NHL.

**Methodology/Principal Findings:**

The effect of BM-MSC was analyzed in two *in vivo* models of disseminated non-Hodgkin's lymphomas with an indolent (EBV^−^ Burkitt-type BJAB, median survival = 46 days) and an aggressive (EBV^+^ B lymphoblastoid SKW6.4, median survival = 27 days) behavior in nude-SCID mice. Intra-peritoneal (i.p.) injection of MSC (4 days after i.p. injection of lymphoma cells) significantly increased the overall survival at an optimal MSC∶lymphoma ratio of 1∶10 in both xenograft models (BJAB+MSC, median survival = 58.5 days; SKW6.4+MSC, median survival = 40 days). Upon MSC injection, i.p. tumor masses developed more slowly and, at the histopathological observation, exhibited a massive stromal infiltration coupled to extensive intra-tumor necrosis. In *in vitro* experiments, we found that: i) MSC/lymphoma co-cultures modestly affected lymphoma cell survival and were characterized by increased release of pro-angiogenic cytokines with respect to the MSC, or lymphoma, cultures; ii) MSC induce the migration of endothelial cells in transwell assays, but promoted endothelial cell apoptosis in direct MSC/endothelial cell co-cultures.

**Conclusions/Significance:**

Our data demonstrate that BM-MSC exhibit anti-lymphoma activity in two distinct xenograft SCID mouse models of disseminated NHL.

## Introduction

Despite the fact that multimodality treatment, including combination chemotherapy, radiation, and target-specific monoclonal antibodies, such as rituximab, can induce high rate of remission in many subtypes of non-Hodgkin's lymphoma (NHL), significant proportions of patients relapse with incurable disease. Thus, effective treatments for NHL remain a serious unmet medical need, also considering that the incidence of NHL continues to rise [1]. The most prevalent forms of NHL are B-cell malignancies, of which follicular lymphoma (FL) and diffuse large B-cell lymphoma (DLBCL) comprise the majority [2]. Burkitt lymphoma is a less prevalent form of NHL characterized by translocation of the c-myc oncogene to the Ig heavy chain promoter/enhancer region [3]. Accumulating evidence has shown that, similarly to solid tumors, the stromal cell component in NHL is not formed by innocent bystanders in the neoplastic process, but it is composed by cell types that might actively influence and promote the growth of the adjacent transformed cells [4]–[9]. However, the effect of human bone marrow (BM) mesenchymal stem cells (MSC), which are considered the stromal progenitor stem cells within the BM, on the growth of tumoral cells is controversial.

MSC are usually isolated from the adherent mononuclear fraction of BM aspirates, can proliferate for many passages in culture and have several properties that make them an attractive choice as cell therapeutic agents. In fact, they are relatively nonimmunogenic, although the mechanism of their immune privilege is not well understood and is a subject of intense study [10]–[12]. Because of these properties, MSC exhibit considerable therapeutic potential in degenerative diseases [13], [14]. On the other hand, regarding their potential therapeutic use in neoplastic diseases, some studies have suggested that adoptively transferred MSC could favor tumor engraftment and progression *in vivo* [9], [12], [15]. The deleterious effects could derive from different MSC characteristics. Indeed, MSC specifically migrate toward sites of active tumorigenesis, where they could integrate the specialized tumor niche, contribute to the development of tumor-associated fibroblasts and myofibroblasts [16]–[18], stimulate angiogenesis [18]–[20], and promote the growth and drug resistance of both solid tumors and hematological malignancies [18], [21]–[26].

On these bases, in the present study we have investigated the effect of BM-derived MSC administration in two models of disseminated NHL, established by intra-peritoneal (i.p.) injection in nude-SCID mice of EBV^−^ Burkitt-type BJAB and EBV^+^ B lymphoblastoid SKW6.4 cell lines, characterized by an indolent (BJAB) and an aggressive (SKW6.4) behavior. Contrary to our expectation, we found that BM-MSC significantly prolonged the survival of lymphoma bearing xenografts.

## Results

### Characterization of the BJAB and SKW6.4 xenograft models

**Figure 1:**
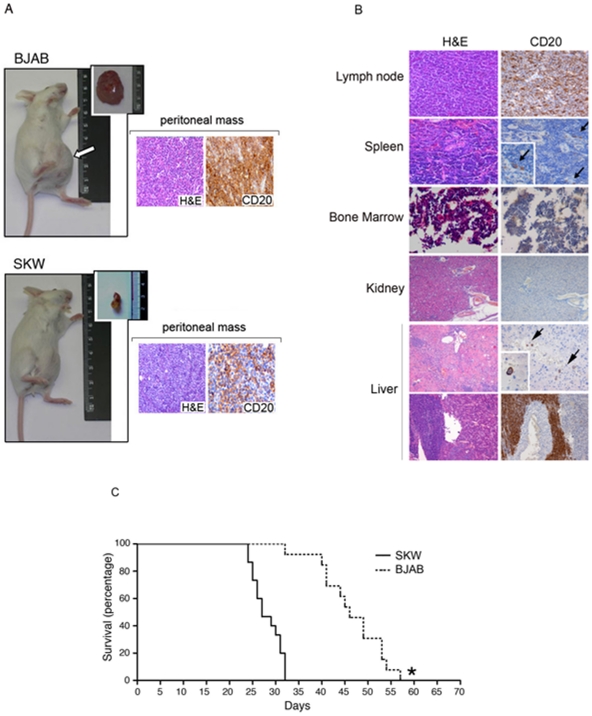
Characterization of the BJAB and SKW6.4 xenograft models. SCID mice were i.p. injected with BJAB or SKW6.4 cells (2×10^6^). **A**, BJAB but not SKW6.4 xenografts were characterized by peritoneal tumors measurable by external observation. Arrow shows the external border of the tumor mass. In the insets, macroscopic appearance of the peritoneal masses and histological H&E and CD20 stainings (original magnification, 20×) are shown. **B**, Histopathological examination of necroptic tissue sections obtained from SKW6.4 xenograft showing absent (kidney and bone marrow), isolated (arrows; spleen and liver) or massive (lymph node and liver) infiltration of CD20^+^ human lymphoid cells. Original magnification, 20×. **C**, Kaplan-Meier survival analysis of the NHL xenograft models. The survival percentage of mice injected with BJAB (n = 15) or SKW6.4 (n = 15) cells was measured from the day of lymphoma cell injection until the day of death. Asterisk, *p*<0.01.

SCID mice were i.p. injected with a pre-determined optimal number (2×10^6^) of lymphoma (BJAB or SKW6.4) cells. BJAB xenografts were characterized by peritoneal tumors, which started to become palpable and measurable by external observation at 18–20 days after injection and steadily progressed until mice death (**Figure 1A**). On the other hand, in SKW6.4 xenografts, a tumor cell mass was hardly palpable at any time point examined. Histopathological examination of the peritoneal masses showed that both BJAB- and SKW6.4-derived tumors have a solid pattern of growth of CD20^+^ lymphoblastoid cells (**Figure 1A**). Variable dissemination of CD20^+^ lymphoblastoid cells was observed in lymph nodes and spleen, while bone marrow and kidneys were usually unaffected (**Figure 1B**). Moreover, both lymphoma-bearing xenografts, and in particular SKW6.4 xenografts, frequently exhibited isolated or massive infiltration of CD20^+^ lymphoblastoid cells in the liver (**Figure 1B**).

Of note, in spite of the bigger peritoneal masses in BJAB with respect to SKW6.4 xenograft mice, the median survival of SKW6.4 xenografts was significantly (p<0.01) shorter (27 days) compared to that of BJAB xenografts (47 days; **Figure 1C**).

### Injection of BM-derived MSC significantly prolongs the survival of both BJAB and SKW6.4 xenografts

**Figure 2:**
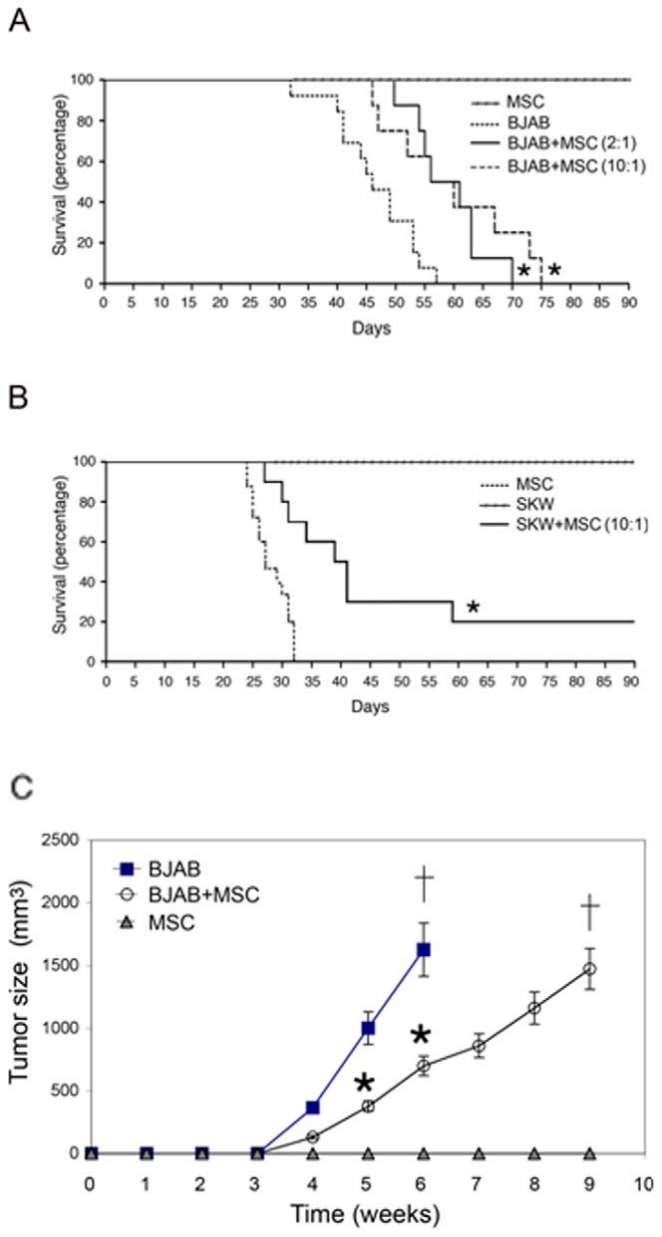
Injection of BM-derived MSC prolonged the survival of BJAB and SKW6.4 xenografts. SCID mice were i.p. injected with either BJAB (n = 10) or SKW6.4 (n = 10) cells and, after 4 days, with MSC at a lymphoma∶MSC ratio of 10∶1. As control, a group of mice (n = 10) was injected with MSC alone. Kaplan-Meier analysis of the NHL xenograft models, comparing the survival of mice injected with BJAB±MSC (**A**) or SKW6.4±MSC (**B**). In BJAB xenograft mice, results obtained in a group of mice (n = 10) injected with MSC at a lymphoma∶MSC ratio of 2∶1 are also shown (**A**). The survival percentage was measured from the day of lymphoma cell injection until the day of death. Asterisk, *p*<0.05 with respect to BJAB or SKW mice. **C**, Peritoneal tumors were measured every week, until animal death, as described in the Material and Methods section.Results are means±SD. Asterisk, *p*<0.05.

To determine the effect of human BM-MSC on the survival of lymphoma xenografts, groups of SCID mice were i.p. injected with either BJAB or SKW6.4 cells and, after 4 days, with MSC at a lymphoma∶MSC ratio of 10∶1. A group of mice was injected with MSC alone, as control. Xenograft mice treated with BJAB+MSC and SKW6.4+MSC showed a significant (p<0.01) increase in survival as compared to the respective BJAB (**Figure 2A**) or SKW6.4 (**Figure 2B**) xenografts. Of note, increasing the amount of injected MSC to a lymphoma∶MSC ratio of 2∶1 did not improve the overall survival of the BJAB xenografts (**Figure 2A**). Moreover, in the BJAB xenograft model, which allowed accurate measurement of the tumor mass by external inspection, we found that the improvement in survival was paralleled by a significant (p<0.05) delay of tumor growth in mice injected with BJAB+MSC with respect to mice injected with BJAB alone (**Figure 2C**).

**Figure 3:**
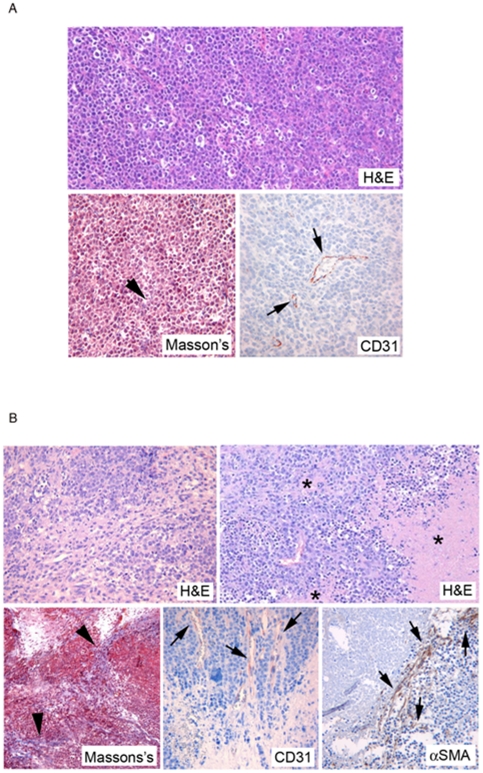
Increased presence of fibrovascular network, necrotic areas and α-SMA^+^ cells in tumor tissues of xenograft lymphomas co-injected with MSC. Sections of peritoneal masses from BJAB (**A**) and BJAB+MSC (**B**) xenograft mice were analyzed after H&E and Masson's trichromic stainings, and immunophenotypical analyses performed with antibodies anti-CD31 or anti-αSMA, as indicated. In H&E staining sections, asterisks indicate small foci of necrosis within tumor masses (**B**). In Masson's trichromic stained sections, collagen fibers are colored in blue (arrowheads) and evidence a thin and inconspicuous stromal meshwork among the solid pattern of the tumor in **A**, or a more diffuse and thick stromal architecture in **B**. CD31^+^ endothelial cells and α-SMA myofibroblast-like cells are colored in brown (arrows in **A** and **B**). Original magnification 20×.

Histopathological examination of the tumoral tissue from the xenograft SCID mice showed strikingly distinct characteristics between peritoneal masses developed upon injection with either lymphoma cells or lymphoma cells+MSC. These analyses were mostly performed on the BJAB xenograft model because of the bigger size of the masses, but similar histopathological characteristics were observed also in the SKW6.4 xenografts. As shown in **Figure 3A** **,** BJAB tumors exhibited a solid pattern of growth with an inconspicuous stromal meshwork, confirmed by Masson's trichrome staining, and few intra-tumor vessels, as detected by CD31 immunohistochemical staining, an histopathological situation reminiscent of that observed in the majority of human NHL [27]. On the other hand, tumoral masses developed in mice co-injected with BJAB and MSC cells showed a completely different aspect, characterized by: areas containing stromal bridges admixed with BJAB cell sheets, as clearly documented by both H&E and Masson's trichromic stainings, and intra-tumor stellate mesenchymal cells often characterized by positivity to α-SMA (**Figure 3B**). Of particular interest was the finding that mice co-injected with BJAB+MSC showed several intra-tumoral foci of necrosis (**Figure 3B**), and BJAB cell sheets with foci of necrosis were often coincident with areas containing α-SMA^+^ stromal cells (**Figure 3B**).

### The *in vitro* co-culture with MSC marginally affects lymphoma cell survival/proliferation while promotes the release of angiogenic cytokines

**Figure 4:**
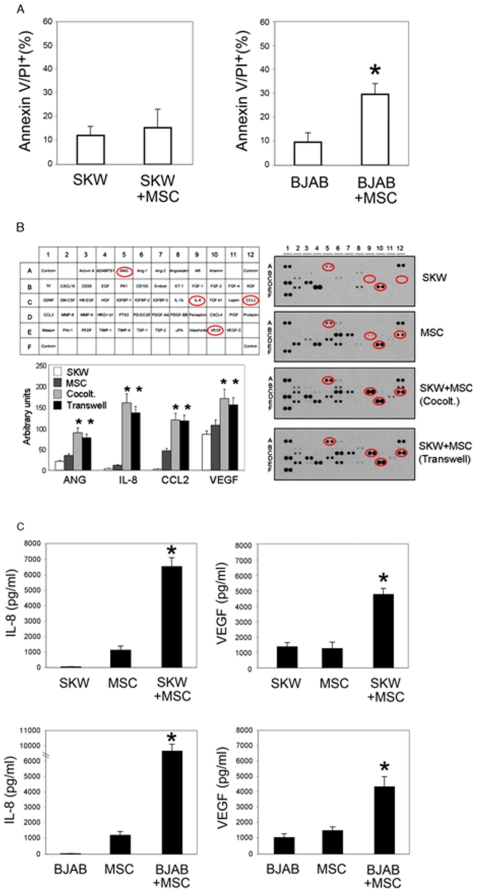
The *in vitro* co-culture with MSC marginally affects lymphoma cell survival and promotes the release of angiogenic cytokines. BJAB and SKW6.4 cells were cultured in the absence or presence of MSC, at a lymphoma∶MSC ratio of 10∶1. **A**, After 96 hours of culture, lymphoma cell apoptosis was analyzed by double Annexin V/PI staining. **B**, Culture supernatants were harvested from: SKW6.4 lymphoma cells alone, MSC alone, SKW6.4/MSC direct co-cultures and SKW6.4/MSC transwell co-cultures. The release of pro-angiogenic cytokines was assessed in the culture supernatants by using a proteome profiler human angiogenesis array. The circles on the membranes highlight four cytokines (angiogenin, IL-8, CCL2 and VEGF) released by MSC, which are significantly up-regulated by the concomitant presence of SKW6.4 cells. Similar results were obtained from three independent experiments and results from one of them are shown. The bar chart reports the results of the densitometric analyses of membranes. Abbreviations of the analyzed cytokines are defined in Table S1. Asterisk, p<0.05. **C**, Levels of IL-8 and VEGF were measured in the culture supernatants of the indicated cultures by ELISA. Data are reported as mean±SD of four independent experiments, each performed in duplicate. Asterisk, p<0.01.

In order to ascertain whether the anti-lymphoma activity of MSC observed *in vivo* was due to a direct inhibitory effect of MSC on lymphoma cells, we have tested whether MSC could modulate the survival/growth of BJAB and SKW6.4 cells in an *in vitro* co-culture system. Cell viability of BJAB cultures showed a moderate but significant decrease (mean±SD: 20±6%, p<0.05), coupled to apoptosis induction, when BJAB were co-cultured in the presence of MSC cells, at a lymphoma∶MSC ratio of 10∶1 (**Figure 4A**). On the other hand, cell viability and apoptosis levels of SKW6.4 cultures, were totally unaffected by the presence of MSC (**Figure 4A**). In addition, in order to compare the release of pro-angiogenic cytokines (**Table S1**), we performed antibody-based protein array analysis of culture supernatants of: i) SKW6.4 lymphoma cells, ii) MSC, iii) SKW6.4/MSC co-cultured in direct contact; iv) SKW6.4/MSC co-cultured in transwell plates. As expected, MSC produced significant levels of several pro-angiogenic cytokines, some of which (angiogenin, IL-8, CCL2 and VEGF) were significantly up-regulated by the concomitant presence of SKW6.4 cells (**Figure 4B**). Both direct and transwell lymphoma/MSC co-cultures were equally effective in promoting the cytokine release. The amounts of IL-8 and VEGF were further measured by ELISA (**Figure 4C**) and the results were consistent with those of the protein array analysis (**Figure 4B**). As shown in **Figure 4C**, it should also be noticed that a significant (p<0.05) increase of the release of IL-8 and VEGF, with respect to MSC alone, was observed both in MSC/SKW6.4 as well as in MSC/BJAB co-cultures.

### MSC induce the migration of endothelial cells and promote endothelial cell apoptosis upon direct MSC/endothelial cell contact

**Figure 5:**
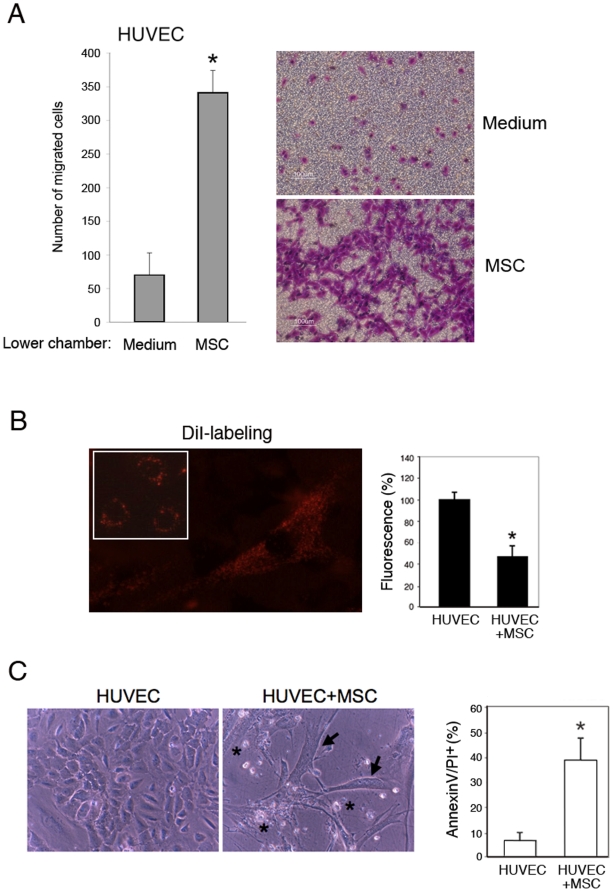
MSC promote the migration of endothelial cells and induce endothelial cell apoptosis upon direct MSC/endothelial cell contact. In **A**, HUVEC migration was assessed in transwell plates toward MSC, seeded (72 h before) in the lower chamber of the transwell plates, vs control medium. 10× magnification photographs of representative stained filters are shown; darker areas are due to higher cell density. The average number of migrating cells per field was assessed by counting at least four high-power random fields per filter. Results are mean±SD from three experiments each performed in duplicate. In **B** and **C**, subconfluent HUVEC were seeded in the absence or presence of MSC (endothelial cell∶MSC ratio of 5∶1). Images by fluorescence and phase contrast microscopy show the significant decrease of endothelial cell density upon addition of MSC to the cultures. **B**, Representative images showing red DiI-labeled endothelial cells as they appear in HUVEC cultures (inset), and MSC, which acquire the staining after 4 days of co-cultures with labeled HUVEC. Original magnification 20×. Overall culture fluorescence of HUVEC and HUVEC+MSC was quantified with a fluorescence plate reader. **C**, The presence of dead cells in representative images by phase contrast microscopy is indicated by asterisks. Original magnification 20×. Overall cell apoptosis was evaluated by Annexin V/PI staining. Data in **B** and **C** are mean±SD of three independent experiments, each performed in duplicate. Asterisck, *p*<0.05.

Since MSC release a cocktail of potent pro-angiogenic factors [28]–[30], we have next examined the ability of MSC to drive the migration of endothelial cells. As shown in **Figure 5A**, MSC cultures exerted a potent (p<0.01) pro-migratory activity on endothelial cells, as evaluated in transwell assays. Based on these observations, in the last group of experiments we have investigated the effect of a direct interaction between MSC and endothelial cells. For this purpose, subconfluent HUVEC were seeded in 6-well plates, and the following day, MSC were added at an endothelial cell∶MSC ratio of 5∶1. In a set of experiments, before the addition of MSC, HUVEC were loaded with the carbocyanine fluorichrome DiI [31], taking into account that DiI transfer from different cell types has been described. MSC/HUVEC o-cultures were then monitored every day by morphological examination (under an inverted phase contrast and fluorescence microscope) and quantification of the overall fluorescence in comparison with cultures of only HUVEC (**Figure 5B**). As expected, on day 1, fluorescence was confined to endothelial cells, while MSC were completely negative. Over time we observed events of fluorescence staining diffuse to MSC, indicating that the MSC had merged to the endothelial cell network (**Figure 5B**). In parallel, in MSC/HUVEC co-cultures, we documented a progressive collapse of the endothelial cell monolayer, with the prevalence of dead endothelial cells nearby the MSC and a significant increase of apoptotic cells, quantified by Annexin-V/PI double staining (**Figure 5B**–**C**). Unfortunately, in these sets of experiments we were unable to perform tripartite experiments with the addition also of lymphoma cells, for technical problems mainly due to differences in culture media requirements.

## Discussion

Previous *in vivo* studies have evaluated the effect of MSC in animal models of hematological malignancies confined in subcutaneous gel devices [14], [15], [32]. Taking into account that the majority of NHL mainly progresses as a systemic malignancy, in this study we have developed and described two animal models that allowed us the investigation of the activity of MSC against NHL in xenografts bearing disseminated tumors. Indeed, after i.p. injection, malignant BJAB and SKW6.4 lymphoma cells formed tumor masses, with frequent dissemination to liver and abdominal lymph nodes.

The major finding of the present study is that a single MSC injection, at MSC∶tumor cell ratio as low as 1∶10, significantly prolonged the survival in animals with indolent (BJAB) and aggressive (SKW6.4) lymphomas. This observation was particularly encouraging since it was obtained in disseminated NHL bearing xenografts, that, in our opinion, are more relevant than the subcutaneous NHL models employed in previous studies [14], [15], [32]. Of note, increasing the number of MSC, up to MSC∶tumor cell ratio of 1∶2, did not result in a significant improvement of the therapeutic efficacy of MSC. The injection of MSC delayed the development of the peritoneal tumoral masses of the NHL xenografts, and at necroscopic analysis, tumor specimens developed in the absence or presence of MSC showed significant histological differences, which can be recapitulated as follow: while BJAB- and/or SKW6.4-derived peritoneal masses showed the typical faint capillary network also described in human NHL [27], the presence of MSC promoted a diffuse increase in α-SMA^+^ cell incorporation throughout the stromal compartment at variance of the scant SMA^+^ perivascular pattern predominantly observed in the absence of MSC. Moreover, massive areas of intra-tumoral necrosis were preferentially observed in lymphoma+MSC injected animals and likely account for the reduced tumor masses characterizing these mice with respect to animals injected with lymphoma cells alone.

In the attempt to elucidate the mechanism(s) by which MSC exert anti-lymphoma activity *in vivo*, we have performed a series of *in vitro* experiments in co-culture systems. The results derived from these experiments tend to exclude a significant direct cytotoxic effect of MSC on lymphoma cells, since MSC moderately inhibited the viability of BJAB but did not exhibit any appreciable effect on SKW6.4 cell survival/growth. Of interest, the ability of MSC cultures of secreting high levels of angiogenic cytokines, such as VEGF, IL-8, angiogenin and CCL2, was significantly (p<0.01) enhanced by the concomitant presence of lymphoma cells. Consistently with their ability of release several pro-angiogenic cytokines, MSC potently promoted the migration of endothelial cells in transwell assays. However, when MSC were directly co-cultured with endothelial cells, we observed a significant induction of endothelial cell apoptosis. In this respect, our current findings are in agreement with those of other Authors who have demonstrated that MSC under certain circumstances might exert anti-angiogenic activity in highly vascularized Kaposi sarcomas [33], as well as in normal endothelial cell cultures *in vitro* [34]. Thus, although we were unable to document the relevance of these *in vitro* data in our animal models, our findings suggest the existence of a complex interplay between MSC, lymphoma cells and endothelial cells, characterized by: i) a significant release of pro-angiogenic/pro-migratory cytokines by MSC, which is enhanced by the presence of lymphoma cells; ii) a potent chemotactic activity of MSC on endothelial cells, followed by a cytotoxic activity of MSC on endothelial cells, which required the MSC/endothelial cell contact.

We are also aware that extrapolation of a given animal model to clinically relevant situations should be considered with extreme caution. In particular, we should remember that an important limitation of our study is represented by the fact that the anti-tumoral immune response is not valuable in our SCID model, but likely it is important in the overall effect of MSC on tumor growth. In fact, it has been shown that the immunosuppressive effect of MSC might favor tumor growth in allogeneic animals [12]. Although some controversial data are present on the role of MSC in cancer development and/or therapy [35], [36], several novel conclusions can be summarized from our study: i) the *in vitro* interaction of BM-derived MSC and lymphoma cells poorly predicts the *in vivo* effect of MSC on the survival of xenograft bearing animals; ii) MSC significantly modulate the stromal network of lymphomas *in vivo*, by increasing the number of α-SMA-positive cells and inducing an increase of intratumor necrosis; iii) MSC promote endothelial cell migration *in vitro* followed by endothelial cell death upon MSC/endothelial direct cell contact.

## Materials and Methods

### Cells

SKW6.4 and BJAB cell lines were obtained from DSMZ (Deutsche Sammlung von Mikroorganismen und Zellkulturen GmbH, Braunschweig, Germany) or purchased from the American Type Culture Collection (ATCC, Manassas, VA), and cultured in RPMI-1640 containing 10% fetal bovine serum (FBS; Gibco BRL, Gaithersbrg, MD). Human BM-derived MSC and human umbilical vein endothelial cells (HUVEC) were purchased from Lonza (Walkersville, MD). BM-MSC were routinely cultured in MSC-Growth Medium (MSC-GM, Lonza). HUVEC were grown on 0.2% gelatin-coated tissue culture plates in M199 endothelial growth medium supplemented with 20% FBS, 10 mg/ml heparin, and 50 mg/ml ECGF (all from Lonza) as previously described [37]. In all experiments, MSC and HUVEC were used between the 3^rd^ and 6^th^ passage *in vitro.*

### NHL mouse xenograft models

Female C.B-17 SCID mice (4 weeks-old) were obtained from Charles River Laboratories (Hollister, CA) and were maintained in accordance with the Guide for the Care and Use of Laboratory Animals. Mice were housed in micro-isolator cages with free access to food and water. The procedures involving animals and their care were conducted in accordance with National Institutes of Health Guide for the Care and Use of Laboratory Animals and were approved by the Institutional Animal Care Committee at the University of Trieste (approval number of the Italian Ministry of Health 191/2008-D). In particular, any effort was put to avoid unnecessary pain of the animals. SKW6.4 or BJAB (2×10^6^) cells were harvested, suspended in PBS, and i.p. injected into 6-week-old mice. After 4 days, lymphoma xenograft mice were randomized into groups (at least 10 mice for each group), and dosed i.p. with vehicle (PBS) or MSC (2×10^5^ or 1×10^6^, corresponding at a lymphoma∶MSC ratio of 10∶1 and 2∶1 respectively). In a group of SKW6.4 mice (n = 10), MSC were injected at 12 days post SKW6.4 injection.

Animals were monitored daily for changes in weight, side effects of the treatment or signs of any sickness. Tumor growth was determined by calliper measurements of two orthogonal axes and the tumor volume was calculated by the formula: (π/6)×*a* ^2^×*b*, wherein *a* is the shorter and *b* is the longer axis; the tumor density was assumed to be equal to one. Survival was calculated as the duration of the animal's life span from the inoculation of lymphoma cells until death. Necropsy was carried out to determine macroscopic extent and histological characteristics of the peritoneal masses. In addition, major organs including heart, kidneys, femur (for bone marrow), liver, spleen, nodes were harvested for microscopic examination and to evaluate the pattern of dissemination of engraftment.

### Histopathological and immunophenotypical analysis

Animal specimens were fixed in 10% buffered-formalin solution and embedded in paraffin. For morphological analysis, 4-µm-thick sections were cut from paraffin blocks and stained with hematoxylin-eosin. Immunohistochemistry was performed by the means of the streptavidin-biotin-peroxidase complex method using the following primary mAbs for: CD20, α-SMA and CD31 (Dako, Glostrup, Denmark). 3-3′diaminobenzidine was used as a chromogen (Vector Laboratories, Burlingame, CA). For determination of collagen content, the sections were stained with Masson's trichrome. After the stainings, the slides were examined under a Leica DM2000 optical microscope and microphotographs were taken using a Leica DFC320 digital camera.

### Co-culture experiments

For lymphoma/MSC co-culture *in vitro* experiments, MSC were seeded in 6 well-plates and the following day, either BJAB or SKW6.4 cells were added to the MSC cultures at a lymphoma∶MSC ratio of 10∶1. Co-cultures were carried out for up 96 hours, in lymphoma cell medium. In some experiments, co-cultures were performed by using 24-Transwell plates (3.0 µm, pore size; Corning Costar, Cambridge, MA), with MSC seeded in the lower compartment and lymphoma cells added in the upper compartment.

For endothelial/MSC co-cultures, HUVEC were seeded in 6-well plates and the following day, MSC were added at a HUVEC∶MSC ratio of 5∶1. Co-cultures were carried out for up 5 days in HUVEC medium. In some experiments, before addition of MSC, HUVEC were loaded with the carbocyanine fluorochrome DiI (1,1′dioctadecyl-3,3,3′,3′tetramethylindocarbocyanine perchlorate; Molecular Probes, Invitrogen, Merelbeke, Belgium). DiI is a lipophilic molecule that incorporates in the cell membrane, and has the following spectral characteristics: absorption maximum at 549 nm and an emission maximum at 565 nm. For this purpose, HUVEC were incubated with 50 µg/ml DiI for 2 hours before performing MSC:endothelial cell co-cultures. Morphology of the HUVEC cultures and HUVEC/MSC co-cultures was observed periodically over time under an inverted phase contrast and fluorescence microscope and the overall fluorescence was quantified with a fluorescence plate reader.

### Apoptosis assay

For analysis of apoptosis, cells were double stained with Annexin V-fluorescein isothiocyanate (Alexis Biochemicals, Lausen, Switzerland) and propidium iodide (PI), according to the instructions of the manufacturer, and analyzed using a FACScan flow cytometer (Becton-Dickinson, San Jose, CA), as previously described [38]. To avoid non-specific fluorescence from dead cells, live cells were gated tightly using forward and side scatter. In particular, for HUVEC and MSC/HUVEC cultures, to analyze the degree of apoptosis in the entire cell population, substrate-attached cells were harvested by trypsin treatment and pooled with floating cells for the staining.

### Proteome profiler array and enzyme-linked immunosorbent assays

Culture supernatants were analyzed by using the proteome profiler human angiogenesis array (R&D Systems, Minneapolis, MN), according to the manufacturer's instructions. Briefly, equal amounts of culture supernatants were diluted and mixed with a cocktail of biotinylated angiogenesis-detection antibodies and incubated with the (capture) antibody membranes overnight at 4°C. After washing of unbound material, the membranes were incubated with HRP-conjugated streptavidin. Chemiluminescence was used for signal detection. Staining intensity of dots were determined with ImageQuant software (Molecular Dynamics) and expressed as arbitrary units.

Enzyme-linked immunosorbent assays (ELISA) for IL-8 and VEGF analyses were performed by using commercially available ELISA kits (R&D Systems). Assays were performed in duplicate and according to the manufacturer's instructions. Results were read at an optical density of 450 nm using an Anthos 2010 ELISA reader (Anthos Labtec Instruments, Wals Salzburg, Austria).

### Endothelial cell migration assay

Cell migration assay was performed in 24-well Transwell plates (8.0 µm, pore size) as previously described [39]. MSC were seeded in HUVEC culture medium into the lower chamber of transwell plates and, after 72 hours, 0.25×10^5^ HUVEC per well were added to the top chambers in 100 µl medium. After 5 hours at 37°C, the upper sides of the filters were carefully washed with PBS, and cells remaining on the upper faces were removed with a cotton wool swab. Transwell filters were then fixed and stained using crystal violet. Cells that had migrated (on the bottom side of the filter) were counted using light microscopy. The average number of migrating cells per field was assessed by counting at least four random fields per filter. Each experiment was done in duplicate.

### Statistical analysis

Results from at least three independent experiments are reported as the means±SD and analyzed for statistical significance by the two-tail Student's t-test and Mann-Witney rank-sum test. Analysis of survival data was carried out with GraphPad Prism version 4 (GraphPad Software); in particular differences in survival between treatment groups were calculated using the Kaplan-Meier curve and survival distribution of the treated and control groups was compared using the log-rank test. Differences were considered significant when *p* value was <0.05.

## Supporting Information

Table S1 Human angiogenesis-related proteins and cytokine array coordinates.
(0.09 MB DOC)
