# Supplementary material for: Human Bone Marrow Mesenchymal Stem Cells Display Anti-Cancer Activity in SCID Mice Bearing Disseminated Non-Hodgkin's Lymphoma Xenografts
Source: PLoS One. 2010 Jun 16;5(6):e11140. doi: 10.1371/journal.pone.0011140 (PMC2886845; doi:10.1371/journal.pone.0011140)
Supplement: Table S1 — Human angiogenesis-related proteins and cytokine array coordinates. (0.09 MB DOC) [file pone.0011140.s001.doc]

**Table S1. Human angiogenesis-related proteins and cytokine array coordinates.**

| **Coordinate** | **Target/Control (Alternate Nomenclature)** |
| --- | --- |
| A1 | Positive Control (Control+) |
| A2 | *empty* |
| A3 | Activin A |
| A4 | ADAMTS-1 |
| A5 | Angiogenin (ANG) |
| A6 | Angiopoietin-1 (Ang-1) |
| A7 | Angiopoietin-2 (Ang-2) |
| A8 | Angiostatin/Plasminogen |
| A9 | Amphiregulin (AR) |
| A10 | Artemin |
| A11 | *empty* |
| A12 | Positive Control (Control+) |
| B1 | Coagulation Factor III (TF) |
| B2 | CXCL16 |
| B3 | DPPIV (CD26) |
| B4 | EGF |
| B5 | EG-VEGF (PK1) |
| B6 | Endoglin (CD1105) |
| B7 | Endostatin/Collagen XVIII |
| B8 | Endothelin-1 (ET-1) |
| B9 | FGF acidic (FGF-1) |
| B10 | FGF basic (FGF-2) |
| B11 | FGF-4 |
| B12 | FGF-7 (KGF) |
| C1 | GDNF |
| C2 | GM-CSF |
| C3 | HB-EGF |
| C4 | HGF |
| C5 | IGFBP-1 |
| C6 | IGFBP-2 |
| C7 | IGFBP-3 |
| C8 | IL1β (IL-1F2) |
| C9 | IL-8 (CXCL8) |
| C10 | LAP (TGF-β1) |
| C11 | Leptin |
| C12 | MCP-1 (CCL2) |
| D1 | MIP-1α (CCL3) |
| D2 | MMP-8 |
| D3 | MMP-9 |

| D4 | NRG1-β1 (HRG1-β1) |
| --- | --- |
| D5 | Pentraxin 3 (PTX3/TSG-14) |
| D6 | PD-ECGF |
| D7 | PDGF-AA |
| D8 | PDGF-AB/PDGF-BB |
| D9 | Persephin |
| D10 | Platelet FActor 4 (PF4/CXCL4) |
| D11 | P/GF |
| D12 | Prolactin |
| E1 | Serpin B5 (Maspin) |
| E2 | Serpin E1 (PAI-1) |
| E3 | Serpin F1 (PEDF) |
| E4 | TIMP-1 |
| E5 | TIMP-4 |
| E6 | Thrombospondin-1 (TSP-1) |
| E7 | Thrombospondin-2 (TSP-2) |
| E8 | μPA |
| E9 | Vasohibin |
| E10 | VEGF |
| E11 | VEGF-C |
| E12 | *empty* |
| F1 | Positive Control (Control +) |
| F12 | Negative Control (Control -) |
